# Market Expansion of Caffeine-Containing Products: Italian and Argentinian Yerba Mate Consumer Behavior and Health Perception

**DOI:** 10.3390/ijerph18158117

**Published:** 2021-07-31

**Authors:** Antonella Samoggia, Pietro Landuzzi, Carmen Enriqueta Vicién

**Affiliations:** 1 Department of Agricultural and Food Sciences, University of Bologna, 40127 Bologna, Italy; pietro.landuzzi@studio.unibo.it; 2 Departamento de Economía, Desarrollo y Planeamiento Agrícola, Universidad de Buenos Aires, Buenos Aires C1417DSE, Argentina; cvicien@agro.uba.ar

**Keywords:** mate, tea, caffeine, health, consumer, market, stakeholder, food neophobia scale

## Abstract

Mate is the most consumed beverage in South America. There is interest in expanding yerba mate sales into the old and new markets by promoting its health properties and energizing effects. The research study aims to explore Argentinian and Italian purchasing and consumption behavior and perception of yerba mate. The exploration includes agro-food chain stakeholders’ views, and consumers’ habits, perception, knowledge of yerba mate in relation to other market positioning caffeine-containing products. Data collection includes qualitative method, such as interviews with agro-food chain stakeholders, that is producers, processors, consumers, and quantitative consumer survey. Data collection was carried out in Argentina and in Italy. Results show that in Argentina yerba mate consumption is driven by habit and tradition, and in Italy yerba mate is mostly unknown. Consumers tend to drink yerba mate in Argentina and other caffeine-containing beverages in Italy to socialize, and as source of energy. Consumers have little awareness of yerba mate antioxidant properties. Yerba mate provides the energy of coffee drinking, and the taste and pleasure of tea drinking. Italian consumers’ key challenge to yerba mate drinking is the longer time it takes, compared to the usual espresso. Italians perceive it as an energetic or relaxing beverage, with a consumption experience similar to tea and infusions. There is need to update commercialization strategy of yerba mate in Italy.

## 1. Introduction

Mate is the most consumed beverage in South America. It is the name of the infusion made with yerba mate, that literally means “mate grass” even though it is made from the crushed, dried and toasted leaves of the shrub *Ilex paraguariensis* [1]. Yerba mate is known with various names, that is Paraguayan tea, Brazilian tea, chimarrao, Misiones or Argentinian tea, Green Gold. The plant grows in the northern part of Argentina, in the south of Paraguay and Brazil, and in Uruguay [2]. In these South American countries mate has been drunk for centuries.

Currently, Argentina is the leading mate producer at a global level (54% of green leaves), followed by Brazil (40%) and Paraguay (6%) [3,4]. Since 2009 yerba mate production has increased consistently, except in 2017, when the harvest decreased by −16% as a reaction to the overproduction in the previous year. Yerba mate sales have increased over time, passing from the 253 million kg in 2016, to 262 million kg in 2018. The popularity of yerba mate in Argentina is confirmed by its inclusion in the basic food basket list [4,5]. The recent yerba mate market sales value is USD 1339 MN, and it is expected to reach USD 1650 MN by 2025 [6]. In 2020, only 14% of the total yerba mate processed in Argentina was exported [6]. The rest was consumed nationally [7,8]. The main export destination for yerba mate is Syria, absorbing 79% of the export, followed by Chile (16%), Libya (3%) and the United States of America (2%) [3,4].

Mate consumers tend to drink it every day, several times a day. The consumption habits are similar among all South-American countries’ population groups, no matter the social or economic class of the consumers. There may be country differences in the way consumers drink it. In Argentina consumers tend to prefer it hot and drank with a straw, whereas in Paraguay it is usually consumed cold. Argentines tend to add sugar to attenuate the characteristic bitter taste of yerba mate. Consumers drink mate throughout the day, and in different contexts. People drink mate alone while studying or working, but it is very common to drink it in a social context. Mate consumption is a cultural and historical tradition, and an important form of social interaction. Thus, yerba mate’s health properties are not a key consumption driver.

In South America, the yerba mate market is at a mature stage, and there is limited market expansion capability [9]. Thus, there is a need to also expand the yerba mate market beyond that continent’s boundaries [9]. The Argentinian National Institute of Yerba Mate activated in 2014 a strategic plan for a sustainable yerba mate production aiming to widen its market positioning in South America, and to increase yerba mate consumer awareness in Europe. The strategic plan aims to improve the awareness about yerba mate’s health properties, to modify the perception of just being a “traditional” drink in South America, to ride the increasing interest in healthy beverages and food in Europe, and to exploit the cultural and culinary heritage of tea and infusion consumption in India [10,11,12].

In the last years, the increased interest in healthy beverages is slowly spreading mate worldwide. Recent studies have highlighted mate’s high quantity of polyphenol antioxidant properties, richness in B_1_, B_6_ and C vitamins, and energizing capability. Yerba mate can also act as a weight management ingredient, help increase mental energy, improve one’s mood, benefit the cardiovascular system, offer anti-inflammatory properties and improve allergy symptoms. Yerba mate’s health attributes have helped it gain popularity in North America. According to Drink Insight Network, yerba mate will expand in the European health drink sector in the next five years [12]. Consumers appreciate mate as a tea. There is increasingly positive inclination towards mate tea use in ready-to-drink (RTD) teas, energy drinks, carbonated soft drinks and other soft drinks. Market data confirm that gluten-free and organic are the most popular health claim on new drinks containing yerba mate globally in the last four years. Mate processors therefore have an interest in exploring yerba mate claims and understanding consumers’ attitude towards yerba mate, new especially among health-oriented and not-informed consumers. This would allow them to differentiate their yerba mate products [13].

The potential of yerba mate in the global market is evident by three other emerging phenomena. First, yerba mate has become an emoji, and since December 2019 it can be used in electronic messages within social media, such as Facebook, Instagram and, WhatsApp. Second, the words yerba mate have registered an increasing interest in Google search trends. In 2019, it was googled twice as much compared to 2004, mainly in South-American countries and increasingly in Europe. Third, the yerba mate image has been promoted by celebrities, who were portrayed with a mate gourd in their hands.

Taking into account the interest in expanding yerba mate sales in old and new markets, and the new research findings regarding yerba mate’s nutritional and health characteristics, there is a need to better understand the consumers’ perception of yerba mate and its market positioning in these markets. Thus, the research study focuses on Argentina, a country where consumers are familiar with yerba mate, and Italy, a country with a long-standing tradition of drinking caffeine-containing beverages, such as coffee. Given the research gaps identified, the research question aims are:

- to explore agro-food chain stakeholders’ views on mate market positioning, market expansion, pricing, and opinions on mate consumers’ habits and perception;
- to explore Argentinian and Italian consumers’ yerba mate purchasing and consumption behavior, focusing on consumers’ habits, perception, knowledge of yerba mate, including mate consumption in relation to other caffeine-containing products;
- to understand consumers’ health perception of yerba mate.

The present study includes various sections. First, yerba mate agronomic characteristics, producing countries, and consumption habits and markets are described. The second section presents a literature review of findings on yerba mate purchasing behavior drivers, perceived consumption benefits, and awareness on yerba mate caffeine content. The third section explains the research methodological framework providing primary and secondary data collection approach, and data analysis. The fourth section provides results pointing out Argentinian and Italian consumers’ behavior and perception. The fifth section discusses present research findings against the available literature. Finally, the study provides conclusive remarks and some market management implications.

## 2. Yerba Mate Production, Processing, and Product Types

### 2.1. Yerba Mate Production and Processing

**Figure 1:**
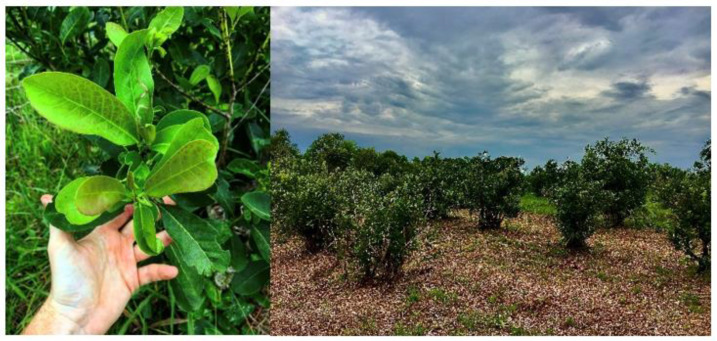
Mate plants and plantations (authors’ photograph).

Yerba mate is a pseudo-edible plant that belongs to the order of Frangulineas and to the family of Aquifoliaceas (Figure 1). The plant can live for more than a century, but the vegetative growth is around 25–30 years. The leave shape depends on the species, and it ranges from 3 to 20 cm. Misiones, an Argentinian region bordering with Paraguay and Brazil, represents the best location for *Ilex paraguariensis* growth. Being a tropical and subtropical plant, it needs high temperatures (annual average 20–23 °C) and at the same time high humidity. The ideal soil for yerba mate is acid clay-sandy soil, rich in potassium and iron, as the one found in northern Argentina [14].

**Figure 2:**
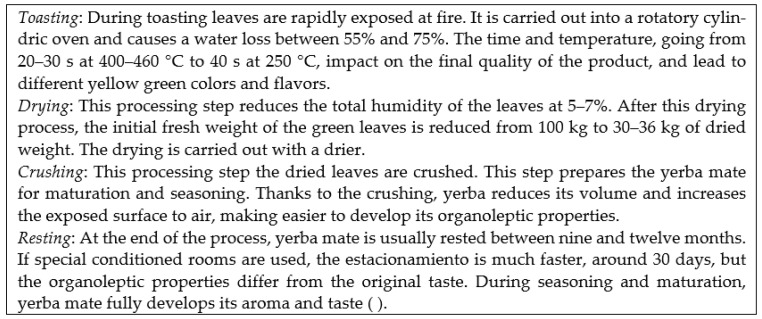
Yerba mate processing steps.

Yerba mate processing is crucial to develop the organoleptic properties of the product. The processing brings aroma, dries the product and makes it homogeneous and adequate for commercialization [14]. The processing is a biological degradation of the vegetative tissue with a strong dehydration driven by smoke and wood combustion [15]. The processing consists in the manual collection of the leaves followed by the steps of toasting (sapecado), drying (secado, barbacuà, carijò), crushing (canchado, despalado) and repose (estacionamiento) [15] (Figure 2).

### 2.2. Yerba Mate Key Stakeholders

The National Institute of Yerba Mate (INYM) is the institution regulating and promoting the value chain of yerba mate. It was established in 2002. INYM aims at supporting the yerba mate stakeholders to monitor the yerba mate market. The INYM develops a price policy for fresh and processed leaves, formulates the Plan Estratégico de la Yerba Mate (PEYM), and manages the Argentinian yerba mate website [3,4]. To promote yerba mate, INYM supports the participation in two important food fairs. The first one is MATEAR, entirely dedicated to yerba mate, and taking place in Buenos Aires. The second one is the Anuga Expo, held in Cologne, Germany.

INYM’s latest data support the fact that in November 2019 there were 12,285 operators in the yerba mate sector, 95% of which were in Misiones, and 3.5% in Corrientes. The vast majority (93%) of yerba mate agro-food chain actors are yerba mate producers. There are 311 processing companies. The yerba mate production system is very polarized, with 11 companies or cooperatives producing and selling 79% of the total yerba mate crop.

In 2018 the leading company was Establecimiento Las Marias S.A. (Gobernador Virasoro, Corrientes, Argentina). Its various brands (Taragüí, Unión, La Merced, Mañanita, Caá Porá) have a 20% market share. It is followed by Cooperativa Agrícola de la Colonia Liebig Ltda (Corrientes, Colonia Liebig, Argentina) (12%) that commercializes the brands Playadito, Yemaypé and Mbareté. Market followers are Establecimiento Diez Hermanos (Apostoles, Misiones, Argentina) and Molinos Río de la Plata (Apostoles, CABA Misiones, Argentina) (9% market share each), La Cachuera SA (Apóstoles, Misiones, Argentina) (8% market share), and Establecimiento Santa Ana (Santa Ana, Misiones, Argentina) (6% market share).

### 2.3. Yerba Mate Composition, Types and Ritual Preparation

**Figure 3:**
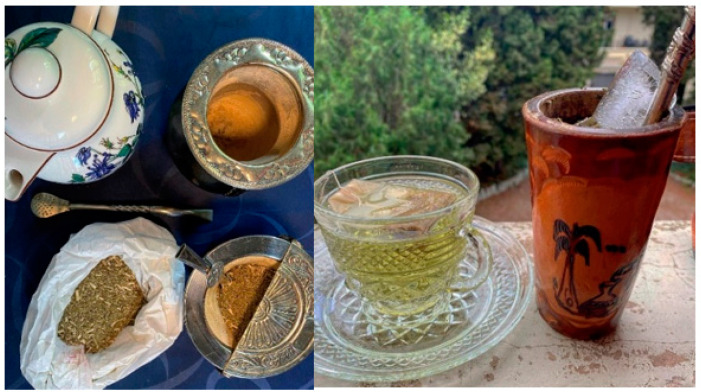
Yerba mate infusion, cocido and terere (authors’ photograph).

The yerba mate format types available for purchase in South America are several (Figure 3). First, the different products have different characteristics based on the combination of three components: powder (polvo), chopped leaves size (hoja) and sticks (palo). The quantity of sticks influences the bitterness, the durability, and the intensity of the yerba mate infusion perception [16,17].

Second, yerba mate format types vary. It is possible to drink mate as dried leaves in the traditional mate recipient or in the mate “cocido” tea bags [16]. The traditional mate preparation uses the mate gourd, the filtering pipe, yerba mate, and hot water. The mate cocido is much easier to drink. It is the same as traditional yerba mate, but placed inside tea bags ready for infusion. The cocido has same nutritional properties as traditional mate, but in a lower quantity. Despite traditionally mate being a hot beverage, there is increasing interest in the cold mate form “terere”, especially in Paraguay and during hot seasons. Terere can be made with cold water and ice, with fresh juice or by adding herbs to the water. Terere has a lower content of polyphenols than hot mate, but higher than a mate cocido [18,19]. To obtain a sweeter drink, consumers add sweetened water or juice to terere. To improve children’s appreciation, mate tea cocido and classic are often mixed with hot milk (80°), coconut, cinnamon or honey.

Third, there is increasing availability of yerba mate mixed with other herbs, for example mint. This product takes the name of “yerba compuesta”, and it is a blend for consumers that do not appreciate the taste of mate, but want to enjoy its benefits [20]. Finally, an increasingly important aspect of yerba mate products is organic certification. It adds sustainable value to the product [17].

## 3. Consumption Behavior of Yerba Mate: Gods’ Healthiest Beverage

### 3.1. Yerba Mate Consumers’ Behavior

In ancient times, there was an historical bond between yerba mate and the indigenous population who used to call it “Gods’ beverage”. Yerba mate “conquered the conquerors” during the colonization, as well as the Jesuit missionaries, thus contributing to its perpetuation in time.

Nowadays yerba mate consumption is deeply rooted in some countries. In Argentina it was selected as the most representative product of the country (38%), even before the asado (37%) meat [21]. However, few studies have analyzed the reasons for mate purchase and consumption. Recent findings support the notion that in Argentina 74% of people drink it out of habit, 64% for its taste, 27% because it keeps company and helps to socialize, and 20% because of the health properties [22]. Research data support the fact that 70% of Argentinians drink mate on a daily basis, and 80% at least once a month. The habit is more frequent among women (84%) than men (75%) [22]. The most purchased yerba mate pack size is the half kilo (53.7%) and the kilo (40.74%). The market offers other sizes too (two kilos and a quarter of a kilo [21]).

A key aspect of yerba mate drinking is its conviviality. Yerba mate preparation and drinking is a ritual to share with other people. According to the ritual, when at least two people share mate, they may share the same straw, and one becomes the cebador, that is the brewer, officiating the ritual and pouring the mate tea to the companion.

The yerba mate diffusion differs across countries. In Syria, mate became popular after Syrian immigration to Argentina during the XIXth Century [3,4]. Now in Syria, as well as in Chile and Uruguay, drinking mate is a tradition, while in other countries, such as Spain, its consumption occurs mainly among Argentinian communities [23].

### 3.2. Yerba Mate Consumption Benefits

Past research has analyzed mate chemical and biological benefits, mainly comparing yerba mate, tea and coffee. The results support the idea that coffee has higher antioxidant properties than tea and mate [24,25]. Other studies support the fact that mate is the hot beverage with the highest antioxidant properties, thanks to its high content of quercetin [26,27].

The different findings on the levels of antioxidant properties takes origin from the different provenance and kinds of yerba mate studied [28]. Uruguayan and Brazilian mate have higher antioxidant effects than the Argentinian one [29].

Other studies have further highlighted yerba mate’s benefits. Mate is effective against obesity [30,31] prevents hearth attacks [32], contributes to avoid the complications of chronic diabetes thanks to its antiglycation action [33], stimulates the nervous system [34], and contributes to intestinal propulsion [26,31,35]. Thus, yerba mate is a pleasant drink, with medicinal properties [36].

There are controversial studies on the relation between mate consumption and the risk of cancer development. Some studies suggest that mate may have carcinogenic effects [37], while others support the idea that mate can prevent it, thanks to its antioxidant properties [38,39].

Finally, in 2014, *Ilex paraguariensis* has been genetically explored, opening the path for molecular marker development, gene mapping, analysis of genetic diversity, and selection breeding in yerba mate [40]. Only in 2020, the academic research defined a lexicon to describe the mate color, aroma, and flavor [41]. The commonly agreed standardization of sensory profile attributes sets the basis of a useful reference for all chain agro-food chain stakeholders.

### 3.3. Consumers and Yerba Mate Caffeine Content

Consumers often appreciate caffeine’s effect on the body and mind awaking properties and mental alertness [41]. Yerba mate contains caffeine (sometimes wrongly called “mateine”). Caffeine intake in Argentina is estimated to be around 288 mg/day per adult [42] and mate, a very common drink, might be the main source of caffeine [43]. Caffeine is the alkaloid (xanthine) responsible for the physiological stimulant effect on the central nervous system. There is increasing public and scientific interest on the health benefits of habitual intake of caffeine-containing beverages. The latest results support the claim that consumption of up to 300–400 mg caffeine per day in healthy adults is not associated with any adverse effects [25,41].

**Figure 4:**
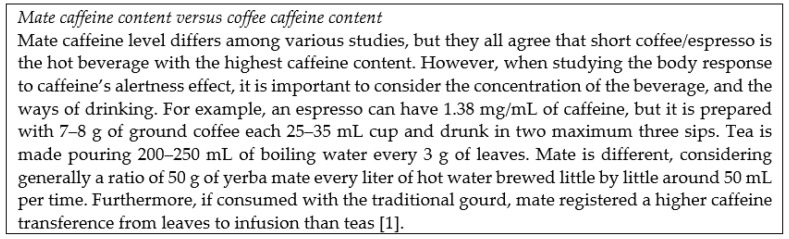
Mate caffeine content.

Estimating yerba mate’s caffeine content is not easy (Figure 4). There have been studies that analyze the chemical composition of mate, with comparisons with other caffeinated beverages. Nevertheless, the results on mate’s caffeine quantity were controversial, possibly for a number of reasons. First, the disparity among caffeine content is due to yerba mate’s elemental percentage. Since caffeine content in leaves is higher than in woody parts, a yerba mate with sticks has a lower caffeine level that one without sticks. Woody sticks lower the caffeine extracting action of hot water. Second, caffeine levels in mate powder depend on the mate origin. Powder from sticks will have lower caffeine ratio than the one deriving from processing leaves. Third, the gap between caffeine levels among studies is due to the different infusion preparation techniques (Table 1).

Studies support that mate’s alertness effect due to the caffeine content is lower than the one obtained from coffee and similar to that of tea. The energy coming from a coffee drink is more intense, but has a shorter duration; while mate has a prolonged effect with no acute peaks.

## 4. Materials and Methods

In order to capture the consumption, production and commercialization features of yerba mate, the methodological framework includes secondary and primary data on the agronomic, commercialization and consumption aspects.

The secondary data include a literature review to inform the data collection. The literature review aimed at exploring the agricultural product characteristics and agro-food chain stakeholder perspectives of yerba mate. The primary data research work includes three phases as described below.

### 4.1. Data Collection

**Figure 5:**
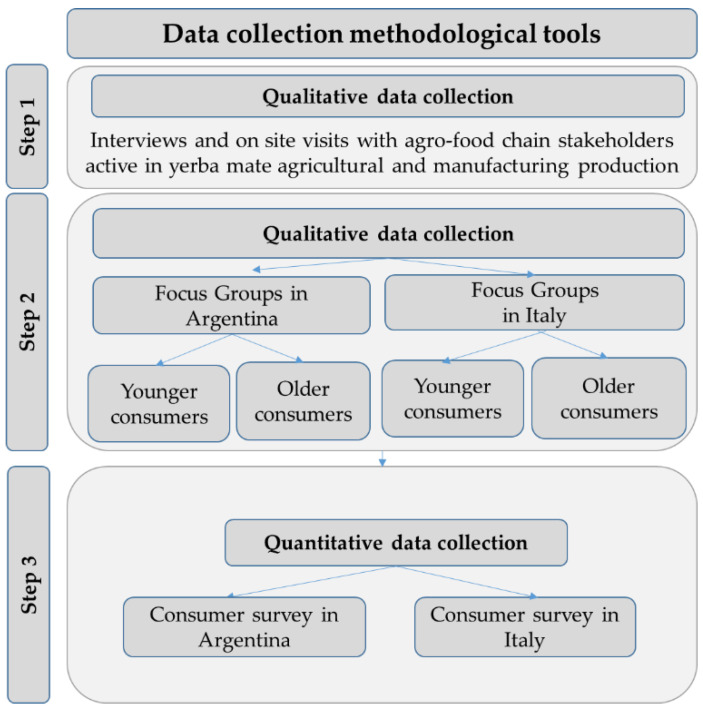
Steps of primary data collection phase.

The primary data collection phase included three steps: (i) interviews with agro-food chain stakeholders and market observations of yerba mate; (ii) focus group interviews with consumers; (iii) a consumer questionnaire survey (Figure 5). Step (i) was carried out in Argentina, and steps (ii) and iii) were carried out in Argentina and in Italy. Data collection was carried out from September 2019 to June 2020 in Argentina, and from January 2020 to November 2020 in Italy. The data collection was based on a detailed literature review carried out to define the content of the focus group and survey interviews (Table 1).

#### 4.1.1. Interviews with Agro-Food Chain Stakeholders and on Site Visits

The qualitative data collection with agro-food chain stakeholders’ interviews and on-site visits was carried out in Argentina and Italy. It aimed at exploring agro-food chain stakeholders’ views on mate market positioning, market expansion, pricing, and opinions on mate consumers’ habits and perception. To capture stakeholders’ understanding of mate market and agro-food chain functioning, the interviews addressed agronomic, processing, marketing aspects of the yerba mate. In particular, in Argentina, the interviews addressed the yerba mate agronomic aspects and the agro-food chain characteristics with specific attention to the yerba mate supply chain organization and commercialization. In Italy, the agro-food chain analysis focused on in person and online visits of the selling outlets, including big retailers and herbalist shops.

The data collection included on-site visits to yerba mate plantations, plant nurseries, yerba mate processor cooperatives, and yerba mate commercial actors. In particular, researchers visited Misiones, the Argentinian region with the highest mate production in the world. Stakeholders allowed to access yerba mate plantations and plant nurseries, and two mate processing centers. The first yerba mate plantation was Yemarì, a natural organic yerba mate cultivation, the second one was Cooperativa Liebig. With its 121 associates this is the biggest yerba mate cooperative in Argentina. Cooperativa Liebig products are commercialized mainly under the brand Playadito that is the most consumed mate brand at a global level. Cooperativa Liebig also has its own processing center to dry and season the leaves, and pack the final product. It is the biggest yerba mate cooperative in Argentina with 36.6 million kilos sold in 2019 [49]. Finally, during the yerba mate expo “Matear”, several yerba mate stakeholders such as entrepreneurs, farmers, and artisans of mate gourds, bombillas and mate bags were interviewed. Matear hosted a few big mate companies like Cruz de Malta and Playadito and many small local producers (Appendix A). There were 25 yerba mate agro-food chain stakeholder interviewees.

#### 4.1.2. Focus Group

**Figure 6:**
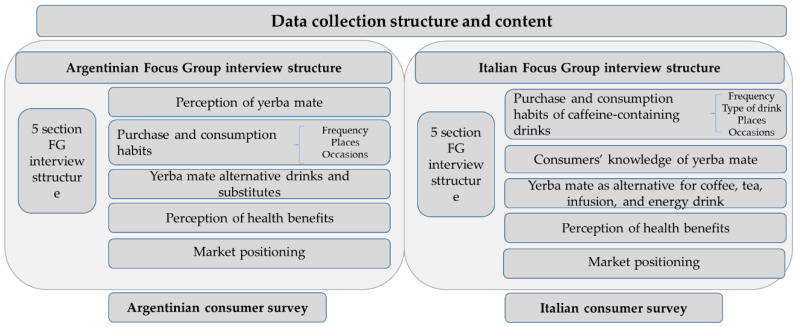
FG data collection structure and content.

Focus groups (FG) aimed at collecting information on consumers’ perception of yerba mate and informing the questionnaire survey data collection. There are studies on Argentinian purchasing and consumption behavior of yerba mate, whereas there are no studies on Italian consumers’ perception of yerba mate. Thus, the FG aimed at consolidating information coming from available studies and setting a basis for the Italian FG analysis (Figure 6).

##### FG Interview Structure

**Figure 7:**
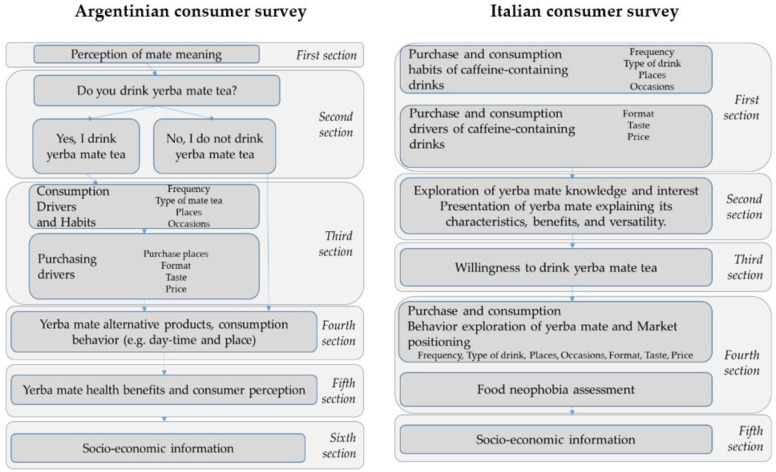
Questionnaire data collection structure and content.

FG interview structure includes five sections (Figure 7). The content differed according to the level of yerba mate knowledge of consumers in the two countries. Argentinian FG include perception of yerba mate, yerba mate purchase and consumption habits, yerba mate alternative drinks, consumers’ knowledge and perception of yerba mate’s health benefits, and yerba mate market positioning. Italian FG included purchase and consumption habits of caffeine-containing drinks to elicit possible knowledge of and experience with yerba mate, exploration of yerba mate as a possible alternative to other caffeine-containing drinks, consumers’ knowledge and perception of yerba mate’s health benefits, and yerba mate market positioning.

FG implementation included a brief introduction about the focus group structure, timing, and purpose. The moderator introduced himself, then each participant followed, including personal information about the age, hobbies, and occupation. The discussion was stimulated with the use of post-its, colors, and word-association.

The Argentinian FG explored the motives for yerba mate drinking, types of yerba mate consumption style (ex. mate, terere or cocido), place and time for yerba mate consumption, authenticity, taste as defined by the culinary tradition, associated food, and possible yerba mate alternatives, mate purchasing places and drivers, brand loyalty, inclination towards yerba mate innovations/experimentations (e.g., flavored), organic, fair-trade, consumption places (e.g., mate bar or coffee shops). Then the moderator introduced the topic of yerba mate alternative drinks. The purpose is to understand the substitutes of yerba mate in consumer’s mind, as well as the conditions, time and place where other beverages represent an alternative to mate. Similarly, Italian consumers presented their habits for caffeine-containing beverages, including coffee, tea, chocolate, yerba mate. They discussed on their consumption styles, place, time, favorite taste, occasions of consumption, organic, and fair-trade attributes. The views exchanges were pushed in the direction of exploring if and how yerba mate may fit within the caffeine-containing beverage consumption.

Then, both countries’ FG explored consumers’ knowledge of yerba mate health benefits. To elicit feedbacks, Post-it^®^ notes with proven yerba mate properties were shown, and participants are asked to associate them to mate.

Finally, Argentinian and Italian consumers were asked about yerba mate market positioning and willingness to pay a higher price for a health-positioning mate. In particular, in Argentina, consumers shared how to sell yerba mate in a country where it is not familiar. In Italy, consumers expressed their views on how they would market-position yerba mate. Argentinian and Italian consumers expressed their view on what type of value proposition, marketing communication plan content, focus on proposed yerba mate property, and finally whether they would propose yerba mate as an ethnic product, or as a breakfast drink, similar to coffee.

##### FG Interview Implementation

In both countries, the study recruited 12 consumers. FG participants were collected according to the snow-ball method and divided in two groups according to their age. The study excluded people younger than 18 years old. In Argentina, FG inclusion criteria foresaw persons drinking mate at least four times per week, and consumers purchasing yerba mate personally, so to have a homogeneous group of informed yerba mate consumers. In Argentina the two FG included above versus below 30 year-old consumers, and in Italy above versus below 25 year-old consumers. Furthermore, each group presented heterogeneity in participants’ gender, social class and nationality according to a non-discriminatory selection criterion.

The discussions were audio-registered under written authorization by the participants. In Argentina, the FG were carried out in person. As a reward, during the FG refreshments, such as cookies and bakery products, an anonymous mate, and a terere to share were offered. In Italy, the FG had to be carried out online due to the COVID pandemic restrictions.

#### 4.1.3. Survey

The consumer survey was carried out in Argentina and in Italy. The survey structure is based on the literature review findings, and FG outcome. The two structures mirror each other, including questions to capture the different consumers’ perception and experience towards yerba mate in the two countries (Figure 7).

##### Survey Structure

o *Argentina*

The questionnaire structure included various sections. The Argentinian consumer survey’s first section aimed at understanding consumers’ mate perception. It aimed at understanding if it is viewed as a tea, an infusion, or an energy drink. The second section includes a filter question to split the respondents into those who drink mate or not. Mate consumers answer Section 3, whereas respondents not drinking mate skipped to Section 4. The third section focuses on yerba mate purchasing and consumption motives and habits (e.g., consumption frequency; type of mate tea, whether hot or cold, tea bags, energy drink, sweetness, liquid chosen during preparation; time and places for consumption; taste, consumption of strong, smoked, organic types, with/without steam, flavored; purchasing outlets; price; promotions; fair-trade; organic; health benefits; brand). The fourth section aimed at eliciting information on yerba mate’s alternatives, their timing during the day, and place of consumption. The fifth section explored yerba mate experienced consumers’, such as Argentinians, perception of yerba mate’s health benefits. The last section collected information on the socio-demographic profiles of the respondents.

o *Italy*

The Italian consumer survey aimed at exploring Italian consumers’ knowledge and perception of yerba mate, and understanding how yerba mate might fit within current Italian consumption of caffeine-containing beverages. The first section aimed at gathering information on purchasing and consumption habits and drivers of caffeine-containing drinks (e.g., consumption frequency; type of drink; time and places for consumption; taste; purchasing outlets; price; promotion). The second section explored consumers’ knowledge about and interest in yerba mate, preliminary presenting information on its characteristics, benefits and versatility. The third section investigated consumers’ willingness to try yerba mate. The positively inclined consumers answer what is or would be their purchasing and consumption behavior and perceived market positioning towards yerba mate. Consumers were asked about the purchasing outlet type, consumption style, price range, retailer physical shelf, etc.

Finally, the consumers were tested against the food neophobia scale. This scale allows one to understand to what extent consumers are reluctant towards food innovation in general. Food neophobia is a trait that can prevent consumers from trying unfamiliar foods and it measures people’s willingness to try out new food or drinks [59]. Food neophobia is assessed by the Food Neophobia Scale (FNS) that is a widely validated 10-item set of questions (Appendix B). Finally, the last section gathered respondents’ socio-economic information.

The questionnaires had close-ended questions. Answers adopted a 5-point Likert, with scale end values anchored from 1 “nothing” to 5 “a lot”. When a different scale was adopted it is specified in the tables presented in the Results section.

##### Survey Implementation

Data collection in both countries was carried out on online. The questionnaires were distributed through the survey automatically generated QR code, and anonymous link through the Qualtrics survey program.

Argentinian respondents were 150. In Argentina the survey-printed QR code distribution was carried out in subway stations, university premises, and shops. They were distributed in Buenos Aires, Santa Fe and Misiones departments. Moreover, the anonymous link was spread via social media and chat. The Italian respondents were 150, and all gave permission for processing their data. In Italy. the survey-printed QR code distribution and anonymous link was available exclusively online due to the COVID movement restrictions implemented at the time.

At the beginning the questionnaire asked for permission for data elaboration, and explained the purpose of the research. The time required to complete the questionnaire was around 6–7 min. There was no reward.

### 4.2. Data Analysis

Data analysis differed between qualitative and quantitative data. Stakeholder interviews and focus groups data were analysed with thematic content analysis. Issues were grouped based on the main literature review findings and in preparation for quantitative analysis. The thematic content data analysis allowed for direct comparison between the four focus groups. As a result, the analysis identified key themes and conceptual combinations focusing on broad themes, as structured in the thematic discussion draft [61,62]. The information was analyzed to find commonalities among the interviewed stakeholders’ opinions on research issues. Therefore, a comprehensive overview of qualitative data was made to define a broad picture. Interview information was prioritized based on the research questions to filter the amount and sources of data [62,63,64,65]. Survey data were analysed with frequencies, means, standard deviations. Chi-square and Anova analyses were carried out to test the significance of the FNS. Consumers’ FNS score was calculated as in the literature (Appendix B).

### 4.3. Survey Sample

The research survey sample includes around 300 consumers, equally distributed between Argentina and Italy (Table 2). In both countries, there were more men (61% in Argentina and 57% in Italy) than women (39% in Argentina and 43% in Italy). The Argentinian sample includes a higher number of students, compared to Italy, where there were more employed people. The average age was above 30 years-old. The Argentinian sample also included consumers from South-American countries familiar with consuming yerba mate.

## 5. Results

### 5.1. Yerba Mate Stakeholders’ View

Argentinian stakeholders are aware of how consumers perceive yerba mate, and support its perception as is the most loved drink in the country. Consumers from any social class drink and share it in the same way. They believe that yerba mate is more than the energy and taste that it gives. They believe in the social purpose of yerba mate. They think that offering a mate is the way to start a conversation, and to welcome another person in a very personal ritual.

Yerba mate stakeholders are aware that to expand their sales, there is a need to target new markets, such as the Europe, yet, they expressed the need to better understand how to approach foreign markets where yerba mate is not known. Stakeholder interview results support the idea that different stakeholders have different perspectives of the yerba mate expansion strategy, and on the role that key stakeholders may have to support market expansion.

The research results sustain the idea that that the yerba mate market expansion strategy can be approached in various ways. First, big and small companies approach the market differently. Interviewees shared the opinion that big companies guarantee competitive prices, and small producers orient their production to a traditional yerba mate with premium and innovative attributes. They think that both a price and quality differentiation strategies, that is sustainability, organic, naturally cultivated, and fair-trade yerba mate products, may be appreciated in Europe. Second, stakeholders believe that yerba mate may be marketed abroad with an innovation perspective. It could sold as a healthy and superfood, and in new blended mate formulations. These may attract new consumers, and make them familiar with the yerba mate taste. Some stakeholders believe that yerba mate could succeed as a healthier alternative to coffee. Third, yerba mate sales and export managers interviewed during expos tend to focus on the organoleptic properties of yerba mate. They invest on unique tastes obtained with special blends, thanks to special traditional mate, and quality infusions. National stakeholders, such as INYM, aim for possible new yerba mate use as mate-based beverages, like energy drinks.

Stakeholder interviews explored the perceived contribution of INYM in supporting yerba mate market and regulation, promoting its consumption, and protecting farmers and workers of the agri-food chain. Stakeholders highlighted the importance of INYM in establishing the “Corresponsabilidad Gremial” tax. This tax is included in the fixed price of yerba mate fresh or dried leaves. It aims at covering the insurance costs of the workers, and interviewees believe it contributes to support a balanced agri-food chain and market relations development.

Interviewees with Playadito stakeholders supported the importance of the company organization for market development. The cooperative activity includes the agricultural production of yerba mate, the nursery, the transformation centers, the storage, until the final packaging. The cooperative analyses the market and invests in innovations at all chain level, including mechanical pruning, new agronomical techniques, and product quality and standardization. According to interviewees, these allow ensuring good product quality and market stability, appreciated by buyers and necessary for market development. For example, plants grown in their nurseries come from certified seeds. All cooperative members have the same plants, so to ensure a homogeneous final quality product.

The relevance of the plant and agronomic aspects to support market expansion was confirmed by other interviewees. The Instituto Nacional de Tecnologìa Agropecuaria (INTA) manages the first and only center of certified *Ilex paraguariensis* seeds. INTA has different reproduction centers all over Argentina, but the one dedicated to yerba mate is the experimental station of Cerro Azul (Misiones). The seeds produced there are sold to different nurseries. The advantages of certified seeds are high successful explants and shortened initiation of production, with reliable raw material results and, thus, final yerba mate production.

The overarching perspective of the stakeholder interviews findings support that yerba mate production and market is strengthening in two directions: the organic-oriented production, following yerbales tradition with a connection to Nature; the production-oriented cooperative, investing in innovation, mainly aiming to guarantee good quality and high yields. Both models can be relevant for the European market. The first may seek consumers oriented towards food product sustainability, and respectful of the country of origin traditions. The second can support the delivery of a good quality, healthy, and possibly price-oriented product to the European consumer market.

Yerba mate is sold in the Italian market in different formats and outlets. In supermarkets traditional yerba mate is available in ½ kilo packs. Retailers tend to sell only one yerba mate brand, which may differ among retailers. For example, a high-level large retailer sells Taragüí brand, by Establecimiento Las Marias, and another medium-high large retailer offers only Amanda brand from La Cachuera SA. Both brands are among the top five operators of the yerba mate market. Yerba mate packaged in Argentina and sold in Europe, in application of the European law, will have a sticker with the nutritional table. In Italy yerba mate is often sold also in herbal shops, sometimes in smaller format or loose, and based on the consumers’ desired weight.

### 5.2. Focus Group

FG provided insights on consumer perception and habits of yerba mate in Argentina and in Italy.

o *Argentina*

Argentinian consumers’ yerba mate purchase and consumption drivers include habit, source of energy, companionship, and means of socialisaton. Mate is part of Argentinian consumers’ tradition and identity. It is part of their folklore and a ritual deeply rooted in their culture, characterized by a number of product perceptions.

*“Mate is much more than only one thing: habits, personal experiences, energy”*

This quote shows how consumers perceive mate as a beverage deeply rooted in Argentinian consumers’ mind. Argentinian consumers associate yerba mate to the green color and Nature. They believe there is a bound between yerba mate and nature. As consumers pointed out:

*“It is common for me to say ’let’s drink a green’ to invite friends to share a mate”*

Furthermore, mate means sharing a drink experience, as it is a social moment to talk and be in a good mood:

*“Yerba mate keeps me company and I believe it means sharing”*

Similar citations were common during FG discussions and underline how the social aspect of mate is tightly linked to this drink.

Yerba mate is their habitual morning and breakfast drink, although Argentinians drink mate while doing different activities, for example studying, working or eating and chatting

*“It is common to drink mate while I do other activities”*

Consumers pay attention to the quality mate drink, that is linked to the mate leaves’ quality and preparation.

*“I want to make good mate and the temperature and gourd can make the difference”*

Finally, there are further differences between younger and older consumers. Younger consumers give importance to the organoleptic properties of yerba mate that should be tasty and fresh, if drunk as terere. They appreciate it as source of energy and as way to socialize. Younger generations are positively inclined towards alternative kind of mate, that is with sugar, coffee, and as terere. Older participants’ consumption behaviour is driven by habit. Some of them started drinking mate very young, in the milky version. As for younger generations, the other reason for drinking mate is to socialize. Moreover, consumers agree that hot mate is the real mate. Older generation appreciate the traditional bitter taste, its smoke aroma, and are interested in premium and organic yerba mate. Moreover, older consumers associate mate to travelling. Mate represents Argentina when they travel around the world, and a mate is never missing in their cars.

Argentinian consumers agree that mate may be substituted, apart from its socialization effect. The most suitable substitute product is coffee. Both drinks provide energy and are stimulants thanks to their caffeine content, yet users perceive a different type of energy. Coffee provides an immediate and stronger waking up action for a limited time, while yerba mate provides a consistent energy boost for a longer time. Another substitute drink is tea. Mate and tea are similar in the consumption style and taste, and consumers did not mention aspects related to caffeine and energy. Consumers may exchange mate with coffee for breakfast and at work, and with tea in the afternoon or, better, when they feel sick.

Younger and older consumers have different knowledge and perception of yerba mate’s health benefits. Young participants are more aware of yerba mate’s health benefits. In particular, they acknowledge mate’s antioxidant and diuretic properties, and may further motivate its consumption. Older consumers are interested in yerba mate’s benefits on human body, but were generally not aware of them. They stressed that an excessive consumption of yerba mate brings stomach acidity. Lastly, a highly appreciated benefit by both groups is the “full-belly” feeling. They acknowledge it could help being on a diet, if drank without sugar.

Consumers have different views on how to promote yerba mate in a new country. Older consumers think that mate should be positioned as an ethnic food, or as an infusion, similar to green tea, so to motivate new consumers to try it, especially if presented as a healthy tea. Younger consumer suggest commercializing yerba mate similarly to coffee, and advertising it as a cheaper and healthy alternative.

o *Italy*

The Italian participants’ selection criteria included coffee, tea, infusion, and energy drink consumers. First, consumers drink coffee because if gives energy, especially in the morning it is “fundamental to start the day”. Consumers drink coffee because they like the taste and aroma, and as a habit. Coffee drinking also has a socialization aspect, especially at work. Tea and infusion consumers are driven by the taste and perceived healthiness. Consumers use energy drinks to get energy, in the evening and at night, and because they are alcohol-free. Furthermore, younger consumers drink coffee for its caffeine content, and tea and infusions should have a delightful taste. Older consumers are interested in the sustainability of food products. Moreover, they appreciate that coffee is energetic and can be drank quickly, and that infusions and tea are expected to provide health-benefits.

Given consumers’ limited knowledge, yerba mate was introduced to participants explaining its characteristics and way of consumption. The researcher showed a traditional mate container with straw, mate tea bags, and ½ kg yerba mate pack. He explained the production process, the origin of the infusion, and its benefits. Thus, he asked to what extent participants would substitute their habitual drinks with yerba mate. There was a rather positive inclination towards yerba mate, with some conditions.

First, coffee consumers would drink mate to reduce their coffee consumption during the day, but not for breakfast. Drinking coffee is an established habit:

*“Food habits matter and, at times, it is not what you drink, but what you are used to”*

Second, Italian consumers treasure the food taste and appreciate coffee taste. Thus, they should like mate taste:

*“Taste is essential! It is not so much a matter of price or anything, what really counts is that yerba mate needs to be good”*

Third, consumers were attracted by the traditional gourd, but the consumption convenience prevailed. For example, a yerba mate glass bottles, and a cold mate in a thermal bottle would be appreciated. Tea consumers would drink mate from a tea bag. They would be motivated by the yerba mate health benefits. Energy drink consumers were attracted by the sugar-free properties. Consumers generally agreed they may be interested in drinking mate the traditional way. Yet, they believe the time necessary to drink it may be a barrier to become a coffee substitute.

*“I am curious about mate and like the philosophy and lifestyle behind this drink. However, mate does not seem functional and fitting with my life, as it is not easy and fast to drink”*

*“I would appreciate mate if drunk fast, similar to Italian espresso”*

*“To some extent mate is mysterious and I like this”*

Consumers provided a number of insights for yerba mate market positioning in Italy. Consumers support the idea that yerba mate may have two targets, that is younger and older consumers. The first group may include to consumers below 35–40 years old, appreciating the yerba mate traditional consumption ritual. These may be sensitive to the booming hype that footballers and celebrities have created around yerba mate. Younger consumers may appreciate mate for its energetic characteristics. The second group would include consumers older than 40 years old, appreciating yerba mate for its health benefits, drank in the tea bags format, easier to consume and containing less caffeine. Overall, Italian consumers may appreciate sustainable yerba mate. In addition, a market positioning focused on the capability to convey energy and health benefits could be the key to yerba mate success in the European market.

Regarding the commercialization outlets, interviewees agreed on having different places to buy or consume mate. Coffee shops, tearooms, and bars offering ready to drink mate are perceived positively. Young people may appreciate a more traditional way of drinking it with the gourd, while older people may prefer it “easy and fast”. Suggested retailing outlets include herbalist’s shops, offering highly priced mate, and retailers, with better affordable prices. Consumers suggest the best product format is the half kilo, shelf positioned close to teas and infusions, and sold as a healthy alternative to coffee. In this case, mate tea should be priced lower than coffee, and similar to tea. There is agreement that mate may also be marketed as an energy drink.

In conclusion, in Argentina mate consumption is driven by habit and tradition, in Italy it is now known. Younger generations are more inclined, compared to older generations, as well as health-oriented consumers. Yerba mate drinking and other caffeine-containing beverages are driven by socialization, and energy seeking experiences. These are factors with differences among generations. Currently, health benefits are not key for yerba mate drinking. Consumers are generally limitedly aware of its antioxidant properties, yet, health benefits may be valid value propositions for traditional and new yerba mate consumers. Yerba mate satisfies the energy need from the coffee and the taste and pleasure of drinking a tea. Mate could be a healthy alternative to coffee, tea, and infusions, still providing hipster and energetic beverage experience. The most significant challenge for yerba mate expansion in other markets, such as Italy, is the time of the yerba mate drinking experience, longer than the usual espresso.

### 5.3. Survey Results

#### 5.3.1. Argentina

Argentinian consumers consider mate mostly as an infusion (39.7%) or as a tea (30.5%). A few consider it as an energetic drink (13.5%) and a ritual (6.4%). The main drivers of mate consumption are habit (33.0%), socialization (24.4%), and taste (22.6%). Mate is limitedly consumed as source of energy (11.7%) and for health benefits (3.9%).

Yerba mate is drunk regularly, as about half of respondents (44.5%) consume it daily. It is consumed for breakfast (40.0%), in during afternoon breaks (49.4%), and rarely at night because of its caffeine content. It is often consumed at home (39.1%), as well as at work (18.5%), and rarely out-of-home. This finding supports the increasing success of thermos market and mate backpacks. It is not drunk when playing sport, as it is not considered an energy drink. The favorite ways of consumption are the traditional hot mate (71.2%), the cold terere (27.3%), and the tea bag cocido (9.3%). The favorite terere type is with juice (47.5%), with cold water (32.5%), and with herbal flavors (20.0%).

Consumers tend to purchase mate at supermarkets (75.3%), and the favorite formats are the half-kilo (50.8%) and kilo (35.3%) packages. The favorite types are the mild flavor intensity (“suave”) (26.6%) and with sticks (“con palo”) (18.6%). Organic mate (10.0%) and smoked flavor (8.0%) are other important mate attributes. Consumers are not interested in consuming innovative yerba mate drink formulations. Most of them have not tried any alternative use (69.2%), and just a small portion (19.2%) is positively inclined towards innovative mate-based products. The most appreciated yerba mate alternative uses are as ingredient in drink and food recipes (10.0%).

**Figure 8:**
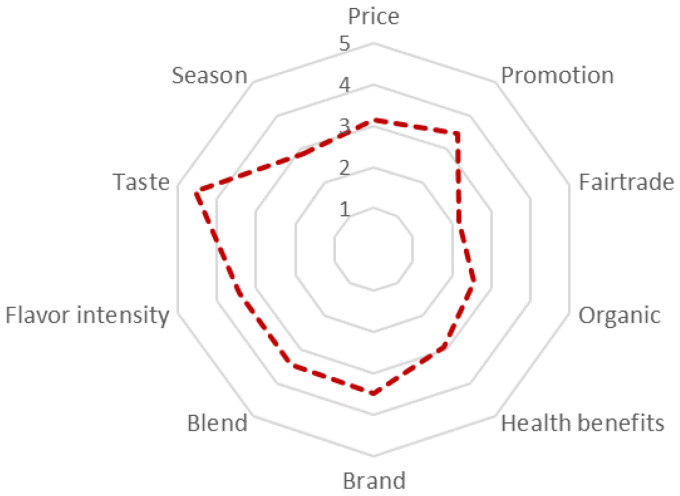
Yerba mate purchasing driving factors.

The key driving factor of yerba mate purchasing is taste (4.53) (Figure 8). The other important elements are brand (3.5), promotion (3.48), blend and intensity (3.43), price (3.14). Fair-trade, organic, health benefits, season have limited importance. Given the importance of the economic attributes, consumers tend to value hedonistic aspects rather than sustainability elements.

Taste is the key element for drinking or for not drinking mate. More than half of consumers do not like its taste. A minority of not mate drinkers supports that sharing the straw is not hygienic (16.7%), and prefer other beverages (16.7%). About a third of consumers do not have a substitute to yerba mate (31.9%), or tend to drink coffee (30.7%), teas (15.1%) and infusions (12.7%). Energy drinks are not valid alternatives to yerba mate.

Coffee is a plausible alternative to mate in the morning (76.6%), at home (39.2%), to some extent at work (26.6%), and in coffee shops (22.8%). Tea is a possible mate substitute in the afternoon (46.7%), mainly at home (67.9%). Infusions may substitute yerba mate (86.4%), especially at night (59.3%). Results on yerba mate health benefits perception support the idea that around one third of consumers think yerba mate has no health benefits (36%). Some consider it an energizer (41.3%), as a help in reducing weight (20.7%), rich in antioxidants (17.3%), and with diuretic properties (10.4%).

#### 5.3.2. Italy

##### Italian Coffee, Tea, Infusion Consumption and Yerba Mate

Italian consumers drink various caffeine-containing drinks, including coffee (41.3%), tea (33.5%), infusions (19.8%), and (4.1%) energy drinks. Drivers of coffee consumption are taste (54%), habit (42%), energizing effects (33.3%), and awakening effect (25.3%). According to coffee consumer behavior, users drink coffee once (25.2%), twice (42.9%), and three times a day (17.7%). Coffee drinking consumption outlets are mostly at home (36.7%), at work (24.2%), and in bars/coffee shops (21.4%). Coffee consumers are willing to try an infusion with energetic properties such as yerba mate.

Tea consumers drink this beverage for its taste (40.3%), relaxation (26.6%), and habit (14.8%). Consumers appreciate the health benefits of tea (10.7%), and energizing effects (7.8%). Italian tea consumers are not focused on innovation. They appreciate classic tea flavor (39.5%), and green teas (22.0%). Black teas (14.5%) and iced teas (lemon 10.5%, peach 11.0%) are possible alternatives. Tea consumption frequency is sporadic (59.8%) and once a day (36.1%). It is mostly drank at home (74.2%), and rarely at work (14.5%) or in coffee shops (8.0%). The vast majority of tea drinkers (85.1%) are willing to try yerba mate, especially if it has healthy properties.

The infusions consumer group is limitedly homogeneous. Consumers drink infusions for relaxation (37.3%), taste (31.8%), and they give importance to healthiness (17.3%), habit (7.3%) and energy (5.5%). Herbal teas were the most common (61.7%), including chamomile (30.9%). Infusion consumption is occasional (73.2%), and daily for a minority of consumers (19.6%). As for teas, infusions are mostly consumed at home (73.6%) and at work (18.1%). Infusion consumers are willing to try an infusion with potential health benefits such as yerba mate (92.3%).

Results support that consumers drink coffee, tea, infusion differently. The various behaviors influence the way they would approach yerba mate, and to what extent yerba mate may replace their favorite drink. Confirming FG outcomes, the survey results support that yerba mate provides the energy of coffee, and the consumption experience of teas and infusions. First, coffee drinkers may appreciate yerba mate if commercialized as an energizer, and tea and infusion drinkers would appreciate a health-oriented claim of yerba mate. Tea and infusion consumers may easily associate yerba mate health benefits herbal teas and chamomile. Second, tea and infusion consumption experience, characterized by medium to long time and use of a high quantity of water, is similar to the yerba mate consumption style. In Italy, coffee is often consumed as espresso that is a fast shot drink.

##### Italian Consumers’ Perception of Yerba Mate

The research provides findings on Italian consumers’ perception of yerba mate properties and ways of consumption (Table 3). Around 66% of respondents had heard about yerba mate. The vast majority of consumers are willing to try it or to drink it again because they appreciate it (85.9%, with 32.8% having already tried it). Results support that 25% of consumers not drinking caffeine-containing beverages would drink yerba mate. All energy drinking consumers are interested in yerba mate. A small minority of coffee (8%) and tea (5%) drinkers would not try yerba mate.

Consumers that did not know yerba mate received a short explanation about yerba mate. Consumers interested in yerba mate expressed their favorite consumption experience. The traditional mate gourd and straw attract the consumers, both hot (42.1%) and cold (11.8%). Consumers also appreciate yerba mate in tea bags (26.7%) and are interested in a yerba mate-based sugar-free healthy energy drink (10.9%), or in capsules for coffee machines (7.7%).

Consumers are positively inclined towards yerba mate, and they would substitute their usual drink with yerba mate, under specific conditions. They should like the taste (42.6%), it should be easy and fast to drink (15.5%), and it should have health benefits (13.2%). Respondents willing to try yerba mate are not very prone to substituting their habitual drink with yerba mate in early morning (20.0%) or after lunch (14.7%), while they would prefer it in the middle of the morning (29.3%) or better in the afternoon (35.8%). The suitable consumption outlet is at home (64.2%), and sometimes at work (27.7%). Consumers expect to purchase yerba mate in different market channels, that is supermarkets (54.0%), herbalist’s shops (38.0%), online (32.7%), specialized shops (23.3%), and in bars/coffee shops ready for consumption (12.7%). When sold in supermarkets, the consumers would search for yerba mate in the tea and infusion aisles (81.1%), close to coffee (11.1%), energy drinks (4.4%), and among health beverages (3.3%). No respondents would look for it among ethnic foods. This is of particular interest, as in Italy it is currently shelf- positioned as an ethnic food.

Finally, the research results support that there is limited relation between food neophobia scale values and willingness to try yerba mate (Table 4). Older consumers tend to be more neophobic (88.1%, mean FNS 1.88), compared to younger consumers (80.8% mean FNS 1.81). ANOVA values are statistically representative. Results support that there is no significant difference among younger and older consumers FNS values cross-analysis with the willingness to try yerba mate.

There is a general positive interest towards yerba mate. It may gain market share from drinks consumed mid-morning and afternoon. That includes coffee, as long as the fast-consumption experience of Italian coffee is ensured. Current commercialization strategy of yerba mate in Italy does not seem to be consistent with Italian consumers’ expectations. They do not perceive it as an ethnic food. It is rather considered an energetic or relaxing and healthy beverage, thus to be found close to tea and infusions.

## 6. Discussion

The research study provides an innovative explorative perspective to the understanding of how to expand yerba mate sales in old and new markets. It provides a comprehensive and preliminary view of agri-food chain actors, from producers to consumers, on how to approach yerba mate production, management and marketing aspects. Past studies limitedly analyzed yerba mate sector from an economic market expansion perspective, including stakeholders, that is small and big producers, national actors, retailing, and consumers [6,19,21]. Other studies focused on yerba mate agronomic and sensory issues [14,15,17,41]. Analyzing yerba mate economic agro-food chain actors’ strategies and consumers’ purchasing and consumption behavior and habits allows to understand what are the possible strategies for market expansion in yerba mate old and new consuming countries. Moreover, the research provides preliminary insights on the capability of valuing the yerba mate nutritional properties as element of competitive advantage for the Argentinian and European market.

### 6.1. Yerba Mate Agro-Food Chain Stakeholders

The current research results support that there are different levels of knowledge, and various visions on how to expand yerba mate sales in new markets. These results confirm the available national and international marketing management research [9,10,13]. Various actors, that is small and big producers, retailers, yerba mate economic, agronomic and management promoting institutions, agree on the need to expand national yerba mate market sales [8,9,11]. Results support that the strategic approaches on how to implement the expansion market lack adequate understanding of the market potential of yerba mate in Argentina, and of the market structure in countries with limited familiarity with yerba mate. These findings confirm past studies [8,9,11].

Interviewees’ feedback confirm that there is no common strategic guideline supporting yerba mate expansion in national and new markets [5,6,7,9]. This leads to various agri-food chain stakeholders’ approaches, mainly related to the producers’ dimensions. Results sustain that yerba mate small agricultural producers tend to revalue old family traditions, and a Nature-friendly yerba mate production process. As pointed out by past studies [66], this addresses their limited environmental awareness and ecological knowledge. As past studies confirm, small agricultural producers are limitedly market oriented, and they believe that rejuvenating old production methods can be positively perceived by the market [3,9]. Big mate producers aim at improving the production yields. They would like to produce three times the currently produced quantity. To further explain past research studies [13,15,66], the current research supports that the low production capability is due mainly to two reasons. First, most of the yerbales (plantations of *Ilex paraguariensis*) are not in good condition. Especially smaller producers have low plant density and inefficient management. Second, old yerbales are often maintained as financial assets, rather than a source of income.

Results confirm that stakeholders have a particular interest about the perspective of internationalizing the yerba mate sales [7,9,11]. They confirm the usefulness of studies that provide a better understanding of new consumers’ needs and wants [17]. The current research show that stakeholders may exploit yerba mate nutritional properties and health benefits. These are promising attributes characterizing an innovative yerba mate market positioning [67].

Argentinian stakeholders need support in interpreting and approaching foreign markets. In particular, these research findings show the mismatch between the commercialization strategies of yerba mate in Italy and consumer perceptions. Yerba mate could be positioned in the tea, infusions and healthy beverages market, rather than in the caffeinated beverage market [48,67].

### 6.2. Yerba Mate Consumers’ Purchasing and Consumption Behavior

Results confirm yerba mate consumption in Argentina is a national dietary tradition driven by taste, habit, and socialization [21,38,46]. There is limited awareness of the mate health benefits, despite its health-benefits were the starting point for mate consumption in past centuries [21,22,23,24,25,26,27,28,29,30,31,32,33,34,36,46]. As highlighted by past studies, women drink mate tea frequently, moreso than males. They are interested in organic certification, more than in fair-trade [20,67,68]. Women value mate’s tea taste, and men are interested in the energetic effects, especially if younger than 40 years old [22]. However, there is a generally limited interest and knowledge about yerba mate’s health benefits. This confirms the opportunity of national stakeholders to promote a comprehensive mate perception. Results support that it is not easy, if possible, to substitute yerba mate with other drinks. For young consumers the most promising drinks may be coffee, for males, and tea and infusions for females. Older consumers would opt for tea [69].

Results confirm that mate tea is not common in Italy, even though a consumer minority, mainly young and women, has tried it. Its current marketing is inconsistent with consumers’ expectations. It has the potential to be a substitute to coffee for its energetic properties, and of tea and infusions for its style of consumption. There are four main resulting aspects. First, consumers do not expect to find mate in retailers’ ethnic food sections where it is currently sold. A different shelf and market positioning could improve the yerba mate perception as Italian consumers tend to associate ethnic foods with unpleasant or risky products. Second, coffee-oriented consumers are limitedly in favor of mate tea, unless their coffee consumption is driven by energy rather than taste. Tea- and infusion-oriented consumers are better inclined towards mate tea. For this group of consumers, taste is a key consumption driver, accompanied with easiness of consumption (e.g., mate tea bags) [70]. Third, as confirmed by other studies, food neophobia results support the idea that younger consumers are better inclined towards new products, compared to older consumers [71]. Fourth, current yerba mate price ranges are perceived as too high, in particular to attract new consumers. Higher prices may be accepted when yerba mate is bought in herbalist shops.

### 6.3. Management Implications

Research results provide a set of evidence-based management implications, mainly focused on yerba mate expansion in the Italian market. These depend on the shopping outlet. First, retailers should define a new shelf positioning. Mate tea should not be placed among ethnic foods, but rather among tea and infusions. Second, mate tea should be sold in tea bag format, to match a consumption style known by consumers. Third, retailers should offer more brands of yerba mate, a wider price range, and new formats, to allow for consumer choice and different purchasing power. Retailers may start commercializing new formats of mate, such as mate capsules, mate-based soft drinks, decaffeinated, chewing-gums, and cocktails [72,73]. Fourth, herbalist shops may offer yerba mate with lower prices, ensure higher service and advice, such as tasting sessions, and include mate sets and accessories in their portfolio to provide a realistic mate experience. In addition, herbalists may offer personalized mate blends, as for teas, to convey the full mate experience. Finally, along with the traditional use, companies may further expand yerba mate use in a number of food products, such as the production of beers, soft drinks, cosmetics, sweets, and functional cheeses as well as other non-traditional uses [74].

## 7. Conclusions

Yerba mate has the potential to become a global market beverage. The current study provides an agro-food chain management perspective on yerba mate, including the agronomic and processing viewpoints, and selling and purchasing strategies. The research provides insights on market expansion of yerba mate, valuing the experience coming from countries with a long tradition in yerba mate production and consumption, and the perceptions and attitudes of new potential markets towards a new beverage.

The study supports that there is need to adjust the yerba mate drinking experience to the expectations of new countries’ consumers. Stakeholders and consumers are positively inclined towards the opportunity of expanding the yerba mate market, both in countries already consuming yerba mate and in countries with low familiarity with the beverage. The study supports that there is limited replicability in yerba mate consumption style in new countries. It supports that valorizing yerba mate nutritional characteristics is an opportunity for both markets. Yerba mate health benefits have the potential to become a new attribute appreciated by nutrition- and health-oriented markets, such as the Europe, and by countries used to appreciate mate for its socializing and energetic properties.

Yerba mate belongs to the caffeine-containing beverage category. Caffeine consumption limits are covered by dietary guidelines across the globe, aiming to set caffeine-intake upper limits to avoid risks. The value of the present study is to confirm preliminary insights on the potentiality of yerba mate as a tea harmonizing energetic effects and health benefits. New market positioning and market expansion require innovative practices involving a number of yerba mate agro-food chain actors in agronomic and economic research to obtain a high-quality raw material, and efficient production and management strategies.

### Limitations and Further Research

The current study is a preliminary exploration on market expansion of a product that is a centuries-old beverage in one country, and a fairly new drink in another country. The food market interest and academic research in yerba mate have expanded in the last years. The fast-increasing interest can make research outcomes rapidly become obsolete. The preliminary nature of the study is mostly due to the country balanced, yet limited sample size. Thus, future studies could stem and further develop from the current study. First, they may study a statistically representative sample. Second, given the importance of taste, they may develop a sensory testing protocol. Finally, they may explore the positioning of yerba mate among health beverages, to assess if yerba mate may become a promising beverage in the European health drink sector in the next five years, as the marketing research agencies foresee for the next decade.

## Figures and Tables

**Table 1:** Literature references of data collection instruments.

Data Collection	Issue	Authors
Argentinian focus group Argentinian questionnaires	Reason for consumption	[21,22,44,45]
Argentinian focus group Argentinian questionnaires	Purchase habits	[5,18,44,46,47,48,49]
Argentinian focus group Argentinian questionnaires Italian focus group Italian questionnaires	Caffeine levels	[26,29,42,43,50,51]
Argentinian focus group Argentinian questionnaires Italian focus group Italian questionnaires	Health properties	[27,28,29,30,34,35,36,37,52,53,54,55,56,57]
Italian focus group Italian questionnaires	Acceptance in new countries	[18,52,58]
Italian focus group Italian questionnaires	Food Neophobia Scale	[59,60]

**Table 2:** Socio-economic characteristics of the respondents.

	Socio-Economic Characteristics	Argentina %	Italy %
Gender	Men	61.1	56.9
	Women	38.9	43.1
	Total	100	100
Employment	Student	58.3	22.3
	Employed	40.6	68.5
	Not active	1.1	9.2
	Total	100	100
Area of living	Main geographical area	85.5 (Argentina)	87.9 (North of Italy)
	Other areas	14.5 (Cile, Paraguay, Brazil, Venezuela, Colombia)	12.1 (Centre, South of Italy)
	Total	100	100
Age	Average	34 year-old	30 year-old

**Table 3:** Italian consumers’ perception of yerba mate properties and ways of consumption.

Would you try yerba mate?	%
Yes, I tried it and I like it	32.8
No, but I would try it	28.9
Yes, but I never tried it and I would like to try	24.2
No, and I would not like to try it	5.5
Yes, I tried it and I do not like it	4.7
Yes, but I never tried it and I would like to try it	3.9
Total	100.0
Consumers (and not consumers) by type of caffeine-containing beverage and willingness to try (or not) yerba mate	
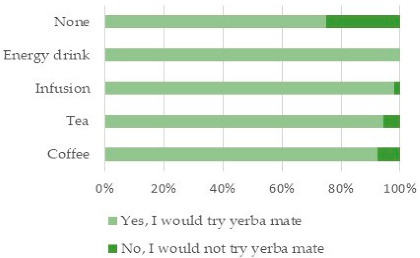	
Would you substitute your coffee, tea, infusion with yerba mate?	%
Yes, it the taste is good	42.6
Yes, if easy to drink	15.5
Yes, if it has health properties	13.2
Yes, absolutely	12.4
Yes, but only in specific times of the day	6.2
No, absolutely	6.2
No, other	1.6
Yes, other	2.3
Total	100.0
When would you drink yerba mate instead of coffee, tea, infusions?	%
Afternoon	35.8
Mid-morning	29.3
Morning for breakfast	20.0
After lunch	14.7
Evening	6.0
Where would you purchase yerba mate?	%
At supermarket	54.0
In herbalist shops	38.0
Online	32.7
Specialized shops	23.3
At the bar-coffee shops	12.7
Other	0.7
Where would you look for yerba mate in your supermarket?	%
Tea and infusions	81.3
Coffee	11.0
Energy drinks	4.4
Healthy beverages	3.3
Total	100.0

Note: Answers with total values are based on single-choice questions; when there is no total values, answers are based on multiple-choice questions.

**Table 4:** Italian consumers’ food neophobia scale values and willingness to try yerba mate.

**FNS Values in Younger and Older Consumers**						
	**Younger %**		**Older %**			
Low FNS values	19.2		11.9			
High FNS values	80.8		88.1			
Total	100.0		100.0			
	Value	Std Dev.	Value	Std Dev.		
**Anova on FNS mean values of younger and older consumers**						
	**Sum of Squares**	**df**	**Mean Square**	**F**	**Sig.**	
Between Groups	0.8	1.0	0.75	4.953	0.028	*
Within Groups	22.7	149.0	0.15			
Total	23.4	150.0				
Mean	1.81	0.4	1.88	0.3		
**FNS Values and Willingness to Try Yerba Mate**						
	**Younger**			**Older**		
	**Yes, I would try** **%**	**No, I would not try** **%**	**Total** **%**	**Yes, I would try** **%**	**No, I would not try** **%**	**Total** **%**
Low FNS values	22	10	21	13	20	14
High FNS values	78	90	79	87	80	86
	100	100	100	100	100	100
Chi-square values	0.338			0.421		

* Significant *p*-value as below 0.05.

## Data Availability

The data that support the findings of this study are available from the corresponding author upon request.

## Appendix A

Yerba mate stakeholder actors interviewed during Matear fair and on-site visits:

- Yerba mate regulator. Director at the National Institute of Yerba Mate (INYM)
- A sommelier de mate. The sommelier processes premium yerba mate dried in a new way to bring out the real taste of the leaves, and sells two kinds of yerba mate: the first one is organic, and the second one is dried with a special vapor technique.
- Mate processor. Specialized in blended mate, their products have high percentage of melted leaves aromatized in different ways. To keep the aroma inside, they have chosen tin cans.
- Mate processor. They produce high quality yerba mate thanks to long maturation period.
- Mate producer and processor. Family-run business oriented on smoked yerba mate product. They only sell one kind of traditional mate pack or mate tea bags.
- Mate processor. This company adds moringa to yerba mate to obtain a superfood. The final product is an infusion very rich in antioxidants.
- Mate producer and processor. The company sells organic yerba mates, easy to find in herbalist shops and in the natural boutiques.
- Mate producer. It is a family run business. The interviewee is one of the farm owners. The yerba mate produced is fully organic and natural yet is still missing the organic seal.
- Mate accessories. They sell handmade backpacks to carry a full mate set.
- Mate accessories. They sell one of the most important mate gourds and straw on the market. They are artisans that, thanks to a focused on-line marketing have reached more than 200,000 followers on Instagram. They have launched the hashtag #MATECHETO (lit. #Coolmate).
- Mate seed certifier. Engineer working in the Argentinian institution for seed certification (INASE) specialized in yerba mate
- Certified mate seed grower. Agronomist in INASE for preservation and evolution of yerba mate plants
- Seed certifier. Director in the Argentina institution for seed certification
- Nursery owner. Expanding yerba mate and other Argentinian indigenous species nursery owner
- Nursery manager. Worker in a yerba mate cooperative taking care of the plantlets exclusively available for the members
- Cooperative member. Stakeholder as member and researcher for innovation in a yerba mate cooperative

## Appendix B

Food Neophobia Scale with numbered items

1. I am constantly trying new drink *
2. I do not trust new drink
3. If I do not know what is in a drink, I will not try it
4. I like drinks from different countries *
5. Ethnic drinks look too weird to try
6. I am afraid to drink things that I have never tried before
7. If I have the chance, I will try new drinks *
8. I am very particular about the beverage I will drink
9. I drink almost everything *
10. I like to try new drinks at the bar *

Items for which scoring is reversed are marked (*) [58].
